# Supplementary material for: MetaRibo-Seq measures translation in microbiomes
Source: Nat Commun. 2020 Jun 29;11:3268. doi: 10.1038/s41467-020-17081-z (PMC7324362; doi:10.1038/s41467-020-17081-z)
Supplement: Supplementary file 10 — Supplementary Data 7 [file 41467_2020_17081_MOESM10_ESM.zip › File2/Confidence_High_Taxonomy/179564_out.krona.html]

Javascript must be enabled to view this page.

members
magnitude
magnitudeUnassigned
count
unassigned
taxon
rank

179564\_out

43

2
43
superkingdom

1239
phylum
43

186801
30
class

30
order
186802

family
1
186803

1952098

SRS104400\_contig\_number\_52178
1
species


SRS150029\_contig\_number\_contig-100\_7410.7411
216572
family
1
21

species
9
1952971

SRS013940\_contig\_number\_contig-100\_41829.41830SRS015431\_contig\_number\_contig-100\_10665.50283SRS015579\_contig\_number\_contig-100\_14613.100088SRS024435\_contig\_number\_contig-100\_5827.257862SRS053356\_contig\_number\_38138SRS053398\_contig\_number\_contig-100\_13815.118092SRS078176\_contig\_number\_10870SRS1041129\_contig\_number\_contig-100\_15841.15842SRS142542\_contig\_number\_20337

459786
genus
11

species
11

SRS016203\_contig\_number\_1220SRS017103\_contig\_number\_5822SRS049773\_contig\_number\_contig-100\_1121.154271SRS050925\_contig\_number\_33719SRS052697\_contig\_number\_contig-100\_1978.218583SRS055017\_contig\_number\_contig-100\_8212.144233SRS075773\_contig\_number\_36084SRS075821\_contig\_number\_17643SRS1041032\_contig\_number\_contig-100\_1312.95168SRS1041141\_contig\_number\_1987SRS146813\_contig\_number\_11642
1945593


SRS054352\_contig\_number\_contig-100\_2012.140796SRS054905\_contig\_number\_13194SRS1041039\_contig\_number\_20985SRS893383\_contig\_number\_12700SRS971276\_contig\_number\_26989
172733
species
5

species
1
1897048

SRS075078\_contig\_number\_40

541000
family
2

292632
1
genus

1897022

SRS075984\_contig\_number\_19609
species
1


SRS150029\_contig\_number\_3743
2292270
species
1

13
species

SRS013521\_contig\_number\_8411SRS049959\_contig\_number\_36812SRS049995\_contig\_number\_19474SRS054905\_contig\_number\_14026SRS098571\_contig\_number\_60502SRS1041039\_contig\_number\_27229SRS1041092\_contig\_number\_14420SRS1041137\_contig\_number\_22657SRS1041145\_contig\_number\_34677SRS144537\_contig\_number\_38911SRS148424\_contig\_number\_33727SRS148817\_contig\_number\_8926SRS149325\_contig\_number\_11689
1263003
